# The importance of maternal insulin resistance throughout pregnancy on neonatal adiposity

**DOI:** 10.1111/ppe.12682

**Published:** 2020-04-30

**Authors:** Rodrigo A. Lima, Gernot Desoye, David Simmons, Roland Devlieger, Sander Galjaard, Rosa Corcoy, Juan M. Adelantado, Fidelma Dunne, Jürgen Harreiter, Alexandra Kautzky‐Willer, Peter Damm, Elisabeth R. Mathiesen, Dorte M. Jensen, Lise‐Lotte T. Andersen, Mette Tanvig, Annunziata Lapolla, Maria G. Dalfra, Alessandra Bertolotto, Urszula Manta, Ewa Wender‐Ozegowska, Agnieszka Zawiejska, David J. Hill, Frank J. Snoek, Judith G. M. Jelsma, Mireille van Poppel

**Affiliations:** ^1^ Institution of Sport Science University of Graz Graz Austria; ^2^ Department of Obstetrics and Gynecology Medizinische Universitaet Graz Graz Austria; ^3^ Western Sydney University Campbelltown New South Wales Australia; ^4^ The Institute of Metabolic Science Addenbrooke’s Hospital Cambridge UK; ^5^ KU Leuven Department of Development and Regeneration: Pregnancy, Fetus and Neonate, Gynaecology and Obstetrics University Hospitals Leuven Leuven Belgium; ^6^ Division of Obstetrics and Prenatal Medicine Department of Obstetrics and Gynaecology Erasmus MC University Medical Centre Rotterdam Rotterdam The Netherlands; ^7^ Institut de Recerca de l´Hospital de la Santa Creu i Sant Pau Barcelona Spain; ^8^ CIBER Bioengineering, Biomaterials and Nanotechnology Instituto de Salud Carlos III Zaragoza Spain; ^9^ Galway Diabetes Research Centre and College of Medicine Nursing and Health Sciences National University of Ireland Galway Ireland; ^10^ Gender Medicine Unit, Endocrinology and Metabolism Department of Internal Medicine III Medical University of Vienna Vienna Austria; ^11^ Departments of Endocrinology and Obstetrics Faculty of Health and Medical Sciences Center for Pregnant Women with Diabetes Rigshospitalet Institute of Clinical Medicine University of Copenhagen Copenhagen Denmark; ^12^ Department of Gynecology and Obstetrics Odense University Hospital University of Southern Denmark Odense Denmark; ^13^ Department of Clinical Research Faculty of Health Sciences Steno Diabetes Center Odense Odense University Hospital University of Southern Denmark Odense Denmark; ^14^ Department of Clinical Research Faculty of Health Sciences University of Southern Denmark Odense Denmark; ^15^ Universita Degli Studi di Padova Padua Italy; ^16^ Azienda Ospedaliero Universitaria – Pisa Pisa Italy; ^17^ Department of Reproduction Poznan University of Medical Sciences Poznan Poland; ^18^ Recherche en Santé Lawson SA Bronschhofen Switzerland; ^19^ Department of Medical Psychology Amsterdam Public Health research institute Amsterdam UMC Vrije Universiteit Amsterdam Amsterdam The Netherlands; ^20^ Department of Public and Occupational Health Amsterdam Public Health Research Institute Amsterdam UMC Vrije Universiteit Amsterdam Amsterdam The Netherlands

**Keywords:** endocrinology, gynaecology, insulin, lipid metabolism, obesity

## Abstract

**Background:**

Although previous studies evaluated the association of maternal health parameters with neonatal adiposity, little is known regarding the complexity of the relationships among different maternal health parameters throughout pregnancy and its impact on neonatal adiposity**.**

**Objectives:**

To evaluate the direct and indirect associations between maternal insulin resistance during pregnancy, in women with obesity, and neonatal adiposity. In addition, associations between maternal fasting glucose, triglycerides (TG), non‐esterified fatty acids (NEFA), and neonatal adiposity were also assessed.

**Methods:**

This is a longitudinal, secondary analysis of the DALI study, an international project conducted in nine European countries with pregnant women with obesity. Maternal insulin resistance (HOMA‐IR), fasting glucose, TG, and NEFA were measured three times during pregnancy (<20, 24‐28, and 35‐37 weeks of gestation). Offspring neonatal adiposity was estimated by the sum of four skinfolds. Structural equation modelling was conducted to evaluate the direct and indirect relationships among the variables of interest.

**Results:**

Data on 657 mother‐infant pairs (50.7% boys) were analysed. Neonatal boys exhibited lower mean sum of skinfolds compared to girls (20.3 mm, 95% CI 19.7, 21.0 vs 21.5 mm, 95% CI 20.8, 22.2). In boys, maternal HOMA‐IR at <20 weeks was directly associated with neonatal adiposity (β = 0.35 mm, 95% CI 0.01, 0.70). In girls, maternal HOMA‐IR at 24‐28 weeks was only indirectly associated with neonatal adiposity, which implies that this association was mediated via maternal HOMA‐IR, glucose, triglycerides, and NEFA during pregnancy (β = 0.26 mm, 95% CI 0.08, 0.44).

**Conclusions:**

The timing of the role of maternal insulin resistance on neonatal adiposity depends on fetal sex. Although the association was time‐dependent, maternal insulin resistance was associated with neonatal adiposity in both sexes.

**Synopsis**

1 **Study question**

How is the synergic relationship of maternal insulin resistance, fasting glucose, triglycerides, and non‐esterified fatty acids with neonatal adiposity in pregnant women with obesity?

2 **What's already known**

Maternal insulin resistance and fasting glucose have a positive association with neonatal adiposity, and the role of maternal triglycerides and non‐esterified fatty acids on neonatal adiposity remains controversial.

3 **What this study adds**

The association between maternal insulin resistance and neonatal adiposity differed depending on the infant sex. While adiposity in boys was associated directly with maternal insulin resistance in the beginning of pregnancy, adiposity in girls was associated with maternal insulin resistance at 24‐28 weeks of gestation via a chain of relationships through fasting glucose, triglycerides, and free fatty acids.

## 1 BACKGROUND

Maternal obesity is associated with neonatal^1^, ^2^, ^3^, ^4^ and childhood adiposity, and childhood obesity^2^, ^5^ is not only associated with a higher chance of obesity, but also premature death and disability in adulthood.^5^ While maternal insulin resistance and fasting glucose have a positive association with neonatal adiposity,^6^, ^7^, ^8^, ^9^, ^10^, ^11^, ^12^, ^13^ the role of maternal triglycerides and non‐esterified fatty acids on neonatal adiposity remains controversial.^6^, ^7^, ^8^, ^11^ It is unclear whether such associations are causal, in the causal pathway or a consequence of the metabolic pathways involved.

Shapiro et al^6^ reported that HOMA‐IR, glucose, but not triglycerides and non‐esterified fatty acids at 24‐32 weeks of gestation mediated the association between maternal pre‐pregnancy body mass index (BMI) and neonatal adiposity. Although this study^6^ provided relevant insights regarding the possible underlying mechanisms in the relationship between maternal metabolic profile and neonatal adiposity, some aspects still require further evaluation. First, it is crucial that the entire pregnancy period is taken into account. Fetal development depends on the maternal metabolic profile throughout the whole pregnancy period,^6^, ^7^, ^8^, ^9^, ^10^, ^11^, ^12^, ^13^ illustrated by the fact that obese women or women who develop gestational diabetes have larger fetuses already by 20 weeks of gestation.^14^, ^15^ Second, there is evidence that neonatal girls might be more insulin resistant than boys,^16^, ^17^ and mechanisms underlying the relationship between maternal metabolism and neonatal adiposity might differ depending on the neonatal sex.^18^

The aim of this investigation was to evaluate the direct and indirect associations between insulin resistance during pregnancy and neonatal adiposity, as measured by sum of skinfolds. In addition, the direct and indirect associations between fasting glucose, triglycerides, non‐esterified fatty acids, and neonatal adiposity were also assessed. This is a novel approach because it assesses the complexity of the relationships among different maternal health parameters throughout pregnancy and its impact on neonatal adiposity. Although the observational design does not allow to assert on causality, it is a valuable approach for generating hypothesis to be tested in clinical trials.

## 2 METHODS

### 2.1 Design and participants

This is a longitudinal, secondary analysis of the DALI study which was a multicentre parallel randomised trial conducted in nine European countries (Austria, Belgium, Denmark, Ireland, Italy, Netherlands, Poland, Spain, and United Kingdom) during 2012‐2015. Pregnant women with a pre‐pregnancy body mass index (BMI) of ≥29 kg/m^2^, before 20 weeks of gestation, with a singleton pregnancy, and aged ≥18 years were invited to participate.^19^ Exclusions included diagnosis with early gestational diabetes mellitus (GDM), pre‐existing diabetes, and chronic medical conditions. The study was prospectively registered as a randomised controlled trial (RCT) on 21 November 2011 (ISRCTN70595832).

### 2.2 Randomisation, masking, and interventions

Using a computerised random number generator, women were randomised to one of the following groups, pre‐stratified for site: (a) healthy eating; (b) physical activity; (c) healthy eating + physical activity; (d) healthy eating + physical activity + vitamin D; (e) healthy eating + physical activity + placebo; (f) vitamin D; (g) placebo; and (h) control. Staff involved with measurements, but not participants, were blinded to the intervention. Since methodology has been extensively described elsewhere,^19^ only variables of interest will be detailed in this manuscript.

### 2.3 Measurements

#### 2.3.1 Neonatal adiposity

Triceps, subscapular, supra‐iliac, and quadriceps skinfolds were measured with a Harpenden skinfold calliper, and values summed. Each skinfold measurement was repeated once, and if a difference of more than 0.2 mm was registered, a third measurement was performed and the average of the three was taken. Time between birth and measurements was registered in hours—neonatal age at the measurement.

#### 2.3.2 Maternal measurements

Maternal height was determined at baseline with a stadiometer (SECA 206; SECA, Birmingham United Kingdom). Women were weighed on calibrated electronic scales (SECA 888 and 877) at baseline (<20 weeks), 24‐28 weeks, and at 35‐37 weeks of gestation. Body mass index (BMI) was calculated as weight in kg divided by the square of height in metres.

After fasting for 10 hours, blood was collected in three different periods during pregnancy (<20, 24‐28, and 35‐37 weeks of gestation). The procedures were identical in all pregnancy periods. All the samples were centrifuged and separated aliquots (1000 or 250 μL) placed in microrack tubes and stored at −20 or −80°C in the central trial laboratory, prior to analysis, in Graz, Austria. The maternal plasma concentrations of fasting glucose, triglycerides, and non‐esterified fatty acids were quantified. For insulin and leptin, commercially available enzyme‐linked immunosorbent assays (ELISAs) were used. Insulin resistance was derived from homeostasis model assessment (HOMA‐IR).^20^

#### 2.3.3 Covariates

Information on possible covariates was collected in the baseline questionnaire or from medical files: intervention groups, maternal age, gestational age during pregnancy (<20, 24‐28 and 35‐37 weeks), maternal ethnicity (European descent and non‐European descent), maternal education (low [<high school], medium [completed high school] and high [higher education]), smoking status at 35‐37 weeks of gestation (yes and no), gestational age at birth (weeks of gestation), and neonatal age (hours).

### 2.4 Statistical analyses

Prevalence, means, standard deviations (SD), medians, and interquartile ranges (IQR) were used to describe the sample. Descriptive mean differences are presented with respective 95% confidence intervals.

Structural equation modelling was performed to evaluate the direct and indirect associations among maternal HOMA index, glucose, triglycerides, non‐esterified fatty acids, and neonatal sum of skinfolds. Direct association refers to the association between two variables, whereas indirect association refers to the association between two variables that is mediated via a third variable. All the standard errors in the analyses were adjusted for the cluster structure of the data (individuals nested within site of recruitment). Table 1 describes the role of each variable in the diagram in detail, including the variables of interest and the adjustments. We have more than 80% statistical power to detect associations with small effect sizes (*f* ^2^ = 0.06) considering the number of neonatal boys (n = 333) and girls (n = 324) evaluated, accounting for the number of exposures and confounding variables in our analysis. All analyses were performed in STATA version 15 for windows (StataCorp LP).

**TABLE 1:** Pathways between all the variables in the analysis

	Outcomes
Exposures	
HOMA‐IR at 13‐20 wk	HOMA‐Index at 24‐28 and 35‐37 wk; Glucose at 13‐20, 24‐28 and 35‐37 wk; Triglycerides at 13‐20, 24‐28 and 35‐37 wk; Non‐esterified fatty acids at 13‐20, 24‐28 and 35‐37 wk; Neonatal fatness
HOMA‐IR at 24‐28 wk	HOMA‐Index at 35‐37 wk; Glucose at 24‐28 and 35‐37 wk; Triglycerides at 24‐28 and 35‐37 wk; Non‐esterified fatty acids at 24‐28 and 35‐37 wk; Neonatal fatness
HOMA‐IR at 35‐37 wk	Glucose at 35‐37 wk; Triglycerides at 35‐37 wk; Non‐esterified fatty acids at 35‐37 wk; Neonatal fatness
Fasting glucose at 13‐20 wk	Glucose at 24‐28 and 35‐37 wk; Neonatal fatness
Fasting glucose at 24‐28 wk	Glucose at 35‐37 wk; Neonatal fatness
Fasting glucose at 35‐37 wk	Neonatal fatness
Triglycerides at 13‐20 wk	Triglycerides at 24‐28, 35‐37 wk; Neonatal fatness
Triglycerides at 24‐28 wk	Triglycerides at 35‐37 wk; Neonatal fatness
Triglycerides at 35‐37 wk	Neonatal fatness
Non‐esterified fatty acids at 13‐20 wk	Non‐esterified fatty acids at 24‐28, 35‐37 wk; Neonatal fatness
Non‐esterified fatty acids at 24‐28 wk	Non‐esterified fatty acids at 35‐37 wk; Neonatal fatness
Non‐esterified fatty acids at 35‐37 wk	Neonatal fatness
Confounders	
Intervention groups	BMI at 13‐20, 24‐28 and 35‐37 wk;
Gestational age	HOMA‐Index at 13‐20, 24‐28 and 35‐37 wk;
Maternal ethnicity	Glucose at 13‐20, 24‐28 and 35‐37 wk;
Maternal education	Triglycerides at 13‐20, 24‐28 and 35‐37 wk;
Maternal age	Non‐esterified fatty acids at 13‐20, 24‐28 and 35‐37 wk;
Smoking status	Neonatal fatness
BMI at 13‐20 wk	BMI at 24‐28 and 35‐37 wk; HOMA‐Index at 13‐20, 24‐28 and 35‐37 wk; Glucose at 13‐20, 24‐28 and 35‐37 wk; Triglycerides at 13‐20, 24‐28 and 35‐37 wk; Non‐esterified fatty acids at 13‐20, 24‐28 and 35‐37 wk; Neonatal fatness
BMI at 24‐28 wk	BMI at 35‐37 wk; HOMA‐Index at 24‐28 and 35‐37 wk; Glucose at 24‐28 and 35‐37 wk; Triglycerides at 24‐28 and 35‐37 wk; Non‐esterified fatty acids at 24‐28 and 35‐37 wk; Neonatal fatness
BMI at 35‐37 wk	HOMA‐Index at 35‐37 wk; Glucose at 35‐37 wk; Triglycerides at 35‐37 wk; Non‐esterified fatty acids at 35‐37 wk; Neonatal fatness
Neonatal age	Neonatal fatness
Gestational age at birth	

### 2.5 Missing data

We used the maximum likelihood for missing values method, which does not exclude a participant in the analysis because of a missing value in one of the variables. Thus, we avoided selection bias in our analysis.^21^ Therefore, all the 657 neonates and their mothers in the study were included in the main analysis.

### 2.6 Ethics approval

We obtained the local ethics committee approvals in all the nine countries, in which the study was conducted. Moreover, written informed consent of all women was acquired.

## 3 RESULTS

In total, 657 neonates (50.7% boys) and their mothers with obesity were included in the study. On average, the mothers were 32.0 (±5.4) years of age. Median gestational age at delivery was 39.7 (IQR: 38.7; 40.9) weeks of gestation. Most mothers perceived themselves as European descent (86.4%). Among the mothers, 55.9% reported a high educational level and 31.8% a medium educational level. Median neonatal age at measurement was 5.0 (IQR: 2.0; 24.0) hours after birth. Boys exhibited a lower sum of skinfolds (mean sum of skinfolds 20.3 mm, 95% CI 19.7, 21.0) compared to girls (mean sum of skinfolds 21.5 mm, 95% CI 20.8, 22.2). Table 2 presents descriptive data on the mothers’ metabolic variables at <20 weeks of gestation according to the sex of the fetus.

**TABLE 2:** Mean and 95% confidence intervals of the mothers’ anthropometric and metabolic variables at <20 wk of gestation according to the sex of the fetus

Maternal variables	<20 wk of gestation			
	Boys		Girls	
	n	Mean (95% CI)	n	Mean (95% CI)
HOMA‐IR ([µU/mL]*[mmol/L])	327	3.0 (2.7, 3.4)	315	3.3 (2.9, 3.6)
Fasting Glucose (mmol/L)	331	4.6 (4.6, 4.6)	320	4.6 (4.6, 4.7)
Triglycerides (mmol/L)	320	1.3 (1.3, 1.4)	307	1.4 (1.3, 1.4)
Non‐esterified fatty acids (mmol/L)	320	0.6 (0.6, 0.7)	307	0.64 (0.6, 0.7)
BMI (kg/m^2^)	333	34.4 (34.0, 34.8)	324	34.5 (34.0, 35.0)

### 3.1 Maternal insulin resistance in relation to neonatal adiposity

In boys, maternal HOMA‐IR at <20 weeks of gestation was directly associated with neonatal sum of skinfolds (β = 0.35 mm, 95% CI 0.01, 0.70). No other associations of maternal HOMA‐IR with neonatal sum of skinfolds in boys were found (see Table 3). In girls, maternal HOMA‐IR was not directly associated with neonatal sum of skinfolds at any time during pregnancy. However, maternal HOMA‐IR at 24‐28 weeks of gestation was indirectly associated with neonatal sum of skinfolds (β = 0.26 mm, 95% CI 0.08, 0.44).

**TABLE 3:** Path coefficients of the direct, indirect, and total association between maternal HOMA‐IR and neonatal sum of skinfolds

Exposures	Neonatal sex	Neonatal sum of skinfolds (mm)		
		Direct association	Indirect association	Total association
HOMA‐IR at < 20 wk	Boys	0.35 (0.01, 0.70)	−0.02 (−0.17, 0.14)	0.34 (0.01, 0.66)
	Girls	0.20 (−0.02, 0.43)	−0.05 (−0.18, 0.08)	0.15 (−0.08, 0.38)
HOMA‐IR at 24‐28 wk	Boys	−0.43 (−0.88, 0.03)	0.09 (−0.05, 0.22)	−0.34 (−0.83, 0.15)
	Girls	0.01 (−0.41, 0.41)	0.26 (0.08, 0.44)	0.26 (−0.09, 0.61)
HOMA‐IR at 35‐37 wk	Boys	0.05 (−0.12, 0.22)	0.05 (−0.03, 0.14)	0.10 (−0.11, 0.31)
	Girls	−0.01 (−0.12, 0.10)	0.07 (−0.01, 0.15)	0.06 (−0.05, 0.17)

Note

Indirect association refers to the association between the exposure (maternal HOMA‐IR at different periods during pregnancy) and the outcome (neonatal sum of skinfolds) which is mediated by all the variables in the pathway (see Table 1 for detailed description).

**FIGURE 1:**
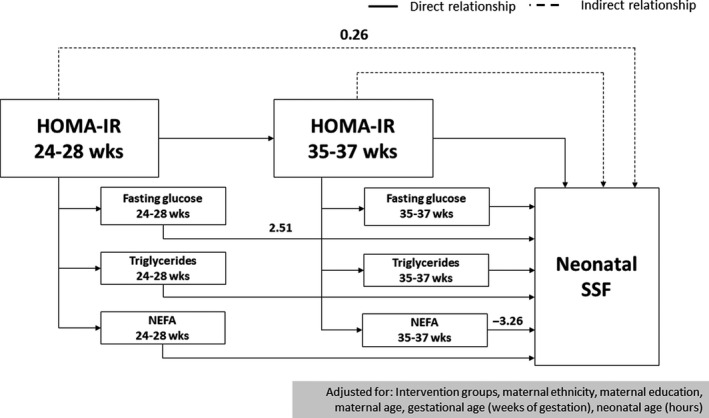
Paths in the association between maternal HOMA‐IR at 24‐28 wk and neonatal sum of skinfolds in girls. Fasting glucose, triglycerides, and non‐esterified fatty acids (NEFA) at 24‐28 wk were directly associated with fasting glucose, triglycerides, and NEFA at 35‐37 wk, respectively. SSF refers to the neonatal sum of skinfolds (mm). Solid lines refer to direct relationships; dashed lines refer to indirect relationships

Figure 1 illustrates the indirect association of maternal HOMA‐IR at 24‐28 weeks of gestation with neonatal sum of skinfolds only in girls because we did not observe an indirect association between maternal HOMA‐IR and neonatal sum of skinfolds in boys. Note that not all the arrows were drawn for clarity. We only drew the most relevant arrows related to the primary and secondary outcomes of the present study. For more details regarding all the relationships tested in our diagram, see Table 1. The indirect association of maternal HOMA‐IR at 24‐28 weeks of gestation with neonatal sum of skinfolds indicates that a higher maternal HOMA‐IR at 24‐28 weeks initiates a chain of relationships through maternal fasting glucose, triglycerides, and NEFA at 24‐28 weeks and HOMA‐IR, fasting glucose, triglycerides, and NEFA at 35‐37 weeks that culminates in a higher neonatal sum of skinfolds (Figure 1). The overall indirect association between maternal HOMA‐IR at 24‐28 weeks and neonatal sum of skinfolds was 0.26 mm (95% CI 0.06, 0.36), and the indirect association between maternal HOMA‐IR at 24‐28 weeks and neonatal sum of skinfolds only via maternal fasting glucose at 24‐28 weeks was 0.21 mm (95% CI 0.06, 0.36), indicating that 81.0% of the indirect relationship between maternal HOMA‐IR at 24‐28 weeks and neonatal sum of skinfolds was mediated via fasting glucose at 24‐28 weeks.

### 3.2 Maternal fasting glucose, triglycerides, and non‐esterified fatty acids in relation to neonatal adiposity

In boys, maternal triglycerides at <20 weeks were indirectly and negatively associated with neonatal sum of skinfolds (β = −0.49 mm, 95% CI −0.97, −0.01). Moreover, triglycerides at 24‐28 weeks of gestation were directly and negatively associated with neonatal sum of skinfolds (β = −1.39 mm, 95% CI −2.58, −0.21). Maternal fasting glucose and NEFA were not associated with neonatal sum of skinfolds in boys at any time during pregnancy (Table 4).

**TABLE 4:** Path coefficients of the direct, indirect, and total association between maternal fasting glucose, triglycerides, non‐esterified fatty acids, and neonatal sum of skinfolds

Exposures, in mmol/l	Gestational age during pregnancy	Outcome: Sum of skinfolds (mm)		
		Boys		
		Total association	Direct association	Indirect association
		β (95% CI)	β (95% CI)	β (95% CI)
Fasting glucose	<20 wk	0.44 (−1.10, 1.98)	−0.31 (−2.06, 1.43)	0.75 (−0.60, 2.11)
	24‐28 wk	0.68 (−1.36, 2.72)	0.09 (−1.86, 2.04)	0.59 (−0.38, 1.57)
	35‐37 wk	1.22 (−0.75, 3.19)	1.22 (−0.75, 3.19)	
Triglycerides	<20 wk	1.12 (−0.59, 2.84)	1.61 (−0.31, 3.54)	−0.49 (−0.97, −0.01)
	24‐28 wk	−0.87 (−1.58, −0.15)	−1.39 (−2.58, −0.21)	0.53 (−0.23, 1.28)
	35‐37 wk	0.78 (−0.26, 1.81)	0.78 (−0.26, 1.81)	
Non‐esterified fatty acids	<20 wk	1.01(−1.27, 3.28)	1.19 (−0.33, 2.71)	−0.18 (−1.92, 1.55)
	24‐28 wk	−1.58 (−5.90, 2.75)	−1.77 (−6.17, 2.62)	0.20 (−0.38, 0.77)
	35‐37 wk	1.32 (−2.56, 5.20)	1.32 (−2.56, 5.20)	

Exposures, in mmol/l	Gestational age during pregnancy	Outcome: Sum of skinfolds (mm)		
		Girls		
		Total association	Direct association	Indirect association
		β (95% CI)	β (95% CI)	β (95% CI)
Fasting glucose	<20 wk	−0.70 (−2.94, 1.54)	−2.52 (−4.86, −0.19)	1.82 (0.85, 2.79)
	24‐28 wk	2.84 (1.38, 4.30)	2.51 (0.80, 4.23)	0.33 (−0.24, 0.90)
	35‐37 wk	0.71 (−0.49, 1.91)	0.71 (−0.49, 1.91)	
Triglycerides	<20 wk	0.80 (−0.30, 1.90)	0.43 (−1.31, 2.17)	0.37 (−0.99, 1.72)
	24‐28 wk	0.01 (−1.47, 1.50)	−0.64 (−2.46, 1.19)	0.65 (−0.44, 1.74)
	35‐37 wk	0.93 (−0.63, 2.49)	0.93 (−0.63, 2.49)	
Non‐esterified fatty acids	<20 wk	1.73 (−0.88, 4.34)	1.47 (−0.38, 3.31)	0.26 (−1.07, 1.60)
	24‐28 wk	2.23 (−1.92, 6.38)	3.02 (−0.99, 7.03)	−0.79 (−1.74, 0.16)
	35‐37 wk	−3.26 (−5.91, −0.61)	−3.26 (−5.91, −0.61)	

Note

Indirect association refers to the association between the exposure and the outcome (neonatal sum of skinfolds) which is mediated by all the variables in the pathway (see Table 1 for detailed description). The empty cells on the indirect relationships between fasting glucose, triglycerides, non‐esterified fatty acids at 35‐37 wk of gestation and neonatal sum of skinfolds are due to the lack of possible indirect relationships to be estimated (there are no intermediate variable between these exposures and the outcome).

In girls, maternal fasting glucose at <20 weeks was inversely and directly associated with neonatal sum of skinfolds (β = −2.52 mm, 95% CI −4.86, −0.19), whereas the indirect association was positive (β = 1.82 mm, 95% CI 0.85, 2.79). Thus, the total association between maternal fasting glucose at <20 weeks and neonatal sum of skinfolds was not significant (β = −0.70 95% CI −2.94, 1.54). Maternal fasting glucose at 24‐28 weeks was directly and positively associated with neonatal sum of skinfolds (β = 2.51 mm, 95% CI 0.80, 4.23). Maternal NEFA at 35‐37 weeks were directly and negatively associated with neonatal sum of skinfolds (β=−3.26 mm, 95% CI −5.91, −0.61). Maternal triglycerides were not associated with neonatal sum of skinfolds in girls at any time during pregnancy (Table 4).

### 3.3 Comment

#### 3.3.1 Principal findings

The primary aim of this investigation was to evaluate the potential pathophysiological pathways underlying the association of maternal insulin resistance with neonatal adiposity in pregnant women with obesity. Since this is a longitudinal study, it is not possible to infer causality in our conclusions. Our results showed that the association between maternal insulin resistance and neonatal adiposity differed depending on the infant sex. While adiposity in boys was associated directly with maternal insulin resistance in the beginning of pregnancy, adiposity in girls was associated with maternal insulin resistance at 24‐28 weeks of gestation via a chain of relationships through fasting glucose, triglycerides, and free fatty acids.

#### 3.3.2 Strengths of the study

Strengths of our study are that we have measures of maternal metabolic parameters at three time points in pregnancy, enabling us to look at longitudinal associations. Data were collected in nine European countries, increasing the external validity of our findings. Importantly, the mechanistic path analysis adds substantial insights on how some of the metabolic parameters during pregnancy in women with obesity could affect neonatal adiposity, although we cannot assert on causality and our model should be tested in clinical trials.

#### 3.3.3 Limitations of the data

Our findings should be interpreted with caution, since women were exposed to different interventions that could influence our results. However, the analyses were adjusted for the intervention component. Another limitation is the lack of normal or overweight women in the study; thus, we can only generalise our findings to Caucasian obese women. However, the prevalence and the increasing rate of obesity in women in reproductive age are alarming and so the complication related to obesity during pregnancy.^22^, ^23^ Furthermore, more direct measures of neonatal adiposity would have been preferred, such as from air displacement plethysmography, although sum of skinfolds provide valid estimates of adiposity in neonates.^24^, ^25^

#### 3.3.4 Interpretation

Regarding the pathophysiological pathways on the relationship between maternal metabolism and neonatal adiposity, Shapiro et al^7^ observed that 21.0% of the association between maternal pre‐pregnancy BMI and neonatal adiposity was mediated by HOMA‐IR and fasting glucose levels, but not by triglycerides or non‐esterified fatty acids.^7^ This is partly in line with our results, although Shapiro et al^7^ included pregnant women with and without obesity whereas we only included pregnant women with obesity. We observed that fasting glucose mediated 81.0% of the association between maternal HOMA‐IR and neonatal adiposity, but only in girls.

The direct association between maternal glucose and neonatal adiposity is well established in the literature.^6^, ^8^, ^9^, ^10^ Results from the Healthy Heart Study^6^, ^7^ and the Hyperglycemia and Adverse Pregnancy Outcome (HAPO) study^26^ demonstrated the importance of maternal glucose levels for neonatal adiposity. In our sample only including pregnant women with obesity, we did not observe any relationship between maternal fasting glucose and neonatal adiposity in boys. In girls, maternal fasting glucose at 24‐28 weeks was associated with neonatal adiposity. No relationship was observed between maternal fasting glucose at <20 weeks or at 35‐37 weeks and neonatal adiposity. We encourage future investigations to evaluate this matter in more depth, with assessment of maternal glucose levels at multiple time points during pregnancy.

The importance of triglycerides and non‐esterified fatty acids levels during pregnancy on neonatal adiposity is not well established.^6^, ^8^, ^11^ When associations are found they are usually positive.^27^ In our sample with pregnant women with obesity, maternal triglycerides were negatively associated with neonatal adiposity in boys at <20 weeks (indirectly) and at 24‐28 weeks (directly). In girls, maternal non‐esterified fatty acids at 35‐37 weeks showed a negative association with neonatal adiposity. Pregnant women with obesity from DALI exposed to motivational sessions on healthy eating presented higher levels of NEFA, and higher levels of NEFA were also found in the cord blood of the offspring when compared to controls.^28^ In addition, maternal diet has been shown to influence triglyceride and free fatty acid levels, even after a period of fasting.^29^ Whether this influenced the association between lipids and neonatal adiposity is unknown. However, there seems to be a complex interaction between maternal glycaemia and lipids in their influence on fetal growth, since maternal lipids were only associated with fetal growth in women with hyperglycaemia but not in those with normal glycaemia.^30^ Whether this is due to differences in insulin resistance and therefore maternal lipolysis needs further study.

Sex differences in the associations between maternal metabolism and neonatal adiposity have been reported previously, but are inconsistent.^16^, ^17^, ^31^ Inconsistencies might be due to study population (ie women with obesity versus with normal weight, with normal glycaemia versus with hyperglycaemia) or due to differences in the timing in pregnancy. Theoretically, male and female fetuses seem to experience different growth strategies in utero.^32^, ^33^ It is not well established yet, but male embryos tend to have more rapid cell divisions compared to females,^34^ generating a more rapid growth.^32^, ^33^ This higher growth rate might explain the higher response to changes in nutrient supply reported in male fetuses.^32^, ^33^ These different growth strategies might result in differences in nutritional needs, at different times in pregnancy and our findings support this concept. However, more systematic assessment of sex differences in the relationship of maternal metabolic parameters with neonatal adiposity is needed.

We evaluated the pathophysiological pathway from maternal insulin resistance, through maternal glucose, triglycerides, and non‐esterified fatty acids to neonatal adiposity in pregnant women with obesity. It indicates again the relevance of the early pregnancy period for neonatal outcomes in boys and in the middle of pregnancy for girls. Although our model explained most of the pathophysiological pathways from insulin resistance to neonatal adiposity in girls, the pathways in boys were still uncertain. A next step would be to investigate the associations of maternal metabolism with cord blood parameters, to confirm the Pedersen‐Freinkel hypothesis that maternal glucose and lipids increase fetal insulin levels and thereby increase fetal fat accretion.^35^

## 4 CONCLUSIONS

Maternal insulin resistance in women with obesity was associated with adiposity in neonates. More specifically, higher insulin resistance in the beginning of pregnancy was related to higher adiposity in boys, whereas this association in girls was observed with insulin resistance in the middle of the pregnancy period. Obstetricians, midwives, and health professionals should be aware of the deleterious impact of maternal insulin resistance on the offspring health and support pregnant women with life style changes (nutrition and physical activity) which could lower insulin resistance during pregnancy and consecutively benefit neonatal body composition.^36^

## References

[1] Goldstein RF , Abell SK , Ranasinha S , et al. Association of gestational weight gain with maternal and infant outcomes. JAMA. 2017;317:2207. 28586887 10.1001/jama.2017.3635 PMC5815056

[2] Gillman MW , Ludwig DS . How early should obesity prevention start? N Engl J Med. 2013;369:2173‐2175. 24224559 10.1056/NEJMp1310577

[3] Sewell MF , Huston‐Presley L , Super DM , Catalano P . Increased neonatal fat mass, not lean body mass, is associated with maternal obesity. Am J Obstet Gynecol. 2006;195:1100‐1103. 16875645 10.1016/j.ajog.2006.06.014

[4] Starling AP , Brinton JT , Glueck DH , et al. Associations of maternal BMI and gestational weight gain with neonatal adiposity in the Healthy Start study. Am J Clin Nutr. 2015;101:302‐309. 25646327 10.3945/ajcn.114.094946 PMC4307203

[5] UNICEF , WHO , World Bank . Joint Child Malnutrition Estimates – Levels and Trends (2018 edition). New York: UNICEF; Geneva: WHO; USA; Washington DC: World Bank; 2018.

[6] Shapiro ALB , Schmiege SJ , Brinton JT , et al. Testing the fuel‐mediated hypothesis: maternal insulin resistance and glucose mediate the association between maternal and neonatal adiposity, the Healthy Start study. Diabetologia. 2015;58:937‐941. 25628236 10.1007/s00125-015-3505-z PMC4393770

[7] Crume TL , Shapiro AL , Brinton JT , et al. Maternal fuels and metabolic measures during pregnancy and neonatal body composition: the healthy start study. J Clin Endocrinol Metab. 2015;100:1672‐1680. 25574704 10.1210/jc.2014-2949 PMC4399301

[8] Friis CM , Qvigstad E , Paasche Roland MC , et al. Newborn body fat: associations with maternal metabolic state and placental size. PLoS ONE. 2013;8:e57467. 23460863 10.1371/journal.pone.0057467 PMC3583865

[9] Lowe LP , Metzger BE , Dyer AR , et al. Hyperglycemia and Adverse Pregnancy Outcome (HAPO) Study: associations of maternal A1C and glucose with pregnancy outcomes. Diabetes Care. 2012;35:574‐580. 22301123 10.2337/dc11-1687 PMC3322718

[10] Aris IM , Soh SE , Tint MT , et al. Effect of maternal glycemia on neonatal adiposity in a multiethnic Asian Birth Cohort. J Clin Endocrinol Metab. 2014;99:240‐247. 24243635 10.1210/jc.2013-2738

[11] Harmon KA , Gerard L , Jensen DR , et al. Continuous glucose profiles in obese and normal‐weight pregnant women on a controlled diet: metabolic determinants of fetal growth. Diabetes Care. 2011;34:2198‐2204. 21775754 10.2337/dc11-0723 PMC3177740

[12] Lowe WL , Lowe LP , Kuang A , et al. Maternal glucose levels during pregnancy and childhood adiposity in the Hyperglycemia and Adverse Pregnancy Outcome Follow‐up Study. Diabetologia. 2019;62:598‐610. 30648193 10.1007/s00125-018-4809-6 PMC6421132

[13] Lowe WL , Scholtens DM , Lowe LP , et al. Association of gestational diabetes with maternal disorders of glucose metabolism and childhood adiposity. JAMA. 2018;320:1005. 30208453 10.1001/jama.2018.11628 PMC6143108

[14] Sovio U , Murphy HR , Smith GCS . Accelerated fetal growth prior to diagnosis of gestational diabetes mellitus: a prospective cohort study of nulliparous women. Diabetes Care. 2016;39:982‐987. 27208333 10.2337/dc16-0160

[15] Macaulay S , Munthali RJ , Dunger DB , Norris SA . The effects of gestational diabetes mellitus on fetal growth and neonatal birth measures in an African cohort. Diabet Med. 2018;35:1425‐1433. 29766563 10.1111/dme.13668

[16] Mitanchez D , Jacqueminet S , Nizard J , et al. Effect of maternal obesity on birthweight and neonatal fat mass: a prospective clinical trial. PLoS ONE. 2017;12:1‐15. 10.1371/journal.pone.0181307 PMC5531500 28750045

[17] Henriksson P , Löf M , Forsum E , Henriksson P , Löf M , Forsum E . Glucose homeostasis variables in pregnancy versus maternal and infant body composition. Nutrients. 2015;7:5615‐5627. 26184296 10.3390/nu7075243 PMC4517020

[18] Lima R , Desoye G , Simmons D , et al. Temporal relationships between maternal metabolic parameters with neonatal adiposity in women with obesity differ by neonatal sex: secondary analysis of the DALI study. Pediatr Obes. 2020;in press. 10.1111/ijpo.12628 PMC7317347 32141687

[19] Jelsma JGM , van Poppel MNM , Galjaard S , et al. DALI: Vitamin D and lifestyle intervention for gestational diabetes mellitus (GDM) prevention: an European multicentre, randomised trial – study protocol. BMC Pregnancy Childbirth. 2013;13:1. 23829946 10.1186/1471-2393-13-142 PMC3710199

[20] Matthews DR , Hosker JP , Rudenski AS , Naylor BA , Treacher DF , Turner RC . Homeostasis model assessment: insulin resistance and beta‐cell function from fasting plasma glucose and insulin concentrations in man. Diabetologia. 1985;28:412‐419. 3899825 10.1007/BF00280883

[21] Maydeu‐Olivares A . Maximum likelihood estimation of structural equation models for continuous data: standard errors and goodness of fit. Struct Equ Model. 2017;24:383‐394.

[22] Ng M , Fleming T , Robinson M , et al. Global, regional, and national prevalence of overweight and obesity in children and adults during 1980–2013: a systematic analysis for the Global Burden of Disease Study 2013. Lancet. 2014;384:766‐781. 24880830 10.1016/S0140-6736(14)60460-8 PMC4624264

[23] Chen C , Xu X , Yan Y . Estimated global overweight and obesity burden in pregnant women based on panel data model. PLoS ONE. 2018;13:e0202183. 30092099 10.1371/journal.pone.0202183 PMC6084991

[24] Schmelzle HR , Fusch C . Body fat in neonates and young infants: validation of skinfold thickness versus dual‐energy X‐ray absorptiometry. Am J Clin Nutr. 2002;76:1096‐1100. 12399284 10.1093/ajcn/76.5.1096

[25] Chen LW , Tint MT , Fortier MV , et al. Which anthropometric measures best reflect neonatal adiposity? Int J Obes. 2018;42:501‐506. 10.1038/ijo.2017.250 PMC5862425 28990589

[26] HAPO Study Cooperative Research Group . Hyperglycemia and Adverse Pregnancy Outcome (HAPO) Study: associations with neonatal anthropometrics. Diabetes. 2009;58:453‐459. 19011170 10.2337/db08-1112 PMC2628620

[27] Barbour LA , Hernandez TL . Maternal lipids and fetal overgrowth: making fat from fat. Clin Ther. 2018;40:1638‐1647. 30236792 10.1016/j.clinthera.2018.08.007 PMC6195465

[28] Harreiter J , Simmons D , Desoye G , et al. Nutritional lifestyle intervention in obese pregnant women, including lower carbohydrate intake, is associated with increased maternal free fatty acids, 3‐β‐hydroxybutyrate, and fasting glucose concentrations: a secondary factorial analysis of the Europea. Diabetes Care. 2019;42:1380‐1389. 31182492 10.2337/dc19-0418

[29] Mudd LM , Holzman CB , Evans RW . Maternal mid‐pregnancy lipids and birthweight. Acta Obstet Gynecol Scand. 2015;94:852‐860. 25912426 10.1111/aogs.12665 PMC4503474

[30] Schaefer‐Graf UM , Meitzner K , Ortega‐Senovilla H , et al. Differences in the implications of maternal lipids on fetal metabolism and growth between gestational diabetes mellitus and control pregnancies. Diabet Med. 2011;28:1053‐1059. 21658120 10.1111/j.1464-5491.2011.03346.x

[31] Wilkin TJ , Murphy MJ . The gender insulin hypothesis: why girls are born lighter than boys and the implications for insulin resistance. Int J Obes. 2006;30:1056‐1061. 10.1038/sj.ijo.0803317 16801943

[32] Lampl M , Jeanty P . Timing is everything: a reconsideration of fetal growth velocity patterns identifies the importance of individual and sex differences. Am J Hum Biol. 2003;15:667‐680. 12953179 10.1002/ajhb.10204

[33] Eriksson JG , Kajantie E , Osmond C , Thornburg K , Barker DJP . Boys live dangerously in the womb. Am J Hum Biol. 2010;22:330‐335. 19844898 10.1002/ajhb.20995 PMC3923652

[34] Mittwoch U . Blastocysts prepare for the race to be male. Hum Reprod. 1993;8:1550‐1555. 8300805 10.1093/oxfordjournals.humrep.a137889

[35] Pedersen J .Diabetes and pregnancy: blood sugar of newborn infants. Nord Med. 1952;25:1049‐1079. 14948109

[36] van Poppel MNM , Simmons D , Devlieger R , et al. A reduction in sedentary behaviour in obese women during pregnancy reduces neonatal adiposity: the DALI randomised controlled trial. Diabetologia. 2019;62:915‐925. 30840112 10.1007/s00125-019-4842-0 PMC6509072

